# Supplementary material for: TRAILS: Tree reconstruction of ancestry using incomplete lineage sorting
Source: PLoS Genet. 2024 Feb 8;20(2):e1010836. doi: 10.1371/journal.pgen.1010836 (PMC10880969; doi:10.1371/journal.pgen.1010836)
Supplement: Attachment — Submitted filename: TRAILS_answer_to_reviewers.pdf [file pgen.1010836.s002.pdf]

Dear editors, dear reviewers,

Thank you for the constructive reviews, which greatly helped improve the manuscript. In summary, we have updated the figures to ease their interpretation, we have included additional simulations to further support our results, and we have included more information in those parts of the text that needed clarification. Below, we include a detailed explanation of the changes made based on the reviews, where reviewer comments are written in black text, while our responses are written in green.

On behalf of all authors,

Iker Rivas-González

General comments from Guest Editor Pier Francesco Palamara and Section Editor Xiaofeng Zhu

The reviewers agree that this is a valuable and well-presented approach for parameter inference in multi-species alignment data and provide some suggestions that could improve the manuscript. They agree on the need to provide additional details on the computational costs of TRAILS compared to previous approaches such as Coal-HMM and a discussion on the scalability to additional lineages or time intervals. Additional comments include suggestions for improving the presentation of results, as well as clarifying the effects of deviations from underlying assumptions on the absence of ILS with the outgroup and the role of admixture/introgression/selection.

We are grateful to the two anonymous reviewers and reviewer Aylwyn Scally for the useful suggestions and comments for improving the manuscript. We have undertaken a careful revision of the manuscript in order to accommodate the comments. In the revised version, we provide details on the computational cost, improve the presentation of results, and clarify the robustness of the model when the underlying assumptions are not fulfilled.

Reviewer's Responses to Questions

Comments to the Authors:
Please note here if the review is uploaded as an attachment.

Reviewer #1: Review of "TRAILS: tree reconstruction of ancestry using incomplete lineage sorting" by Iker Rivas-González, Mikkel H. Schierup, John Wakeley, and Asger Hobolth

In this manuscript, the authors present a novel Coalescent Hidden Markov Model framework TRAILS that is geared towards estimating parameters (speciation times & ancestral population sizes) describing the speciation history of closely related species. In particular, the authors present an application to the human-chimp-gorilla scenario, with orangutan as an outgroup. The method takes as input one genomic sequence for each extant species, including the outgroup. The framework implements the marginal genealogical tree relating the extant lineages at each locus as the hidden state space, where the observed nucleotides are the emissions, and the transition probabilities capture the correlation of genealogies at neighboring loci due to chromosomal linkage and recombination. The method can thus take advantage of incomplete-lineage-sorting (ILS) between the extant species to estimate the parameters.

This is an accurate summary of our framework TRAILS.

With the novel method, the authors improve upon the method CoalHMM that had previously been presented for similar applications. The new method allows the coalescent events of ancestral lineages between speciation events to be classified into a finer number of discrete intervals, rather than just a single interval. The authors demonstrate that this improves estimation accuracy in simulated data. Applying their method to data from Chromosome 1, they were able to estimate parameters for the speciation times that are in agreement with the literature. In addition to parameter estimation, the authors demonstrate that their method can be used to obtain and inspect the posterior distribution of the marginal genealogies, which they show is useful to investigate ILS along the genome, identify potentially introgressed genomic regions, and identify candidates for adaptive genetic variation.

This is an accurate summary of how TRAILS extends CoalHMM, and why it is useful.

The manuscript is well written, presenting the novel method and it's application clearly and accessible. The method is well documented and provided as a software package for convenient use. It is thus a valuable addition to the toolkit of methods to characterize speciation events and related phenomena like ILS and introgression, as well as non-neutral dynamics. I do appreciate the detailed supplement and diagrams therein that make the presentation accessible to non-experts. In addition to some minor comments, I think it would be useful to add some additional simulations and analysis to further support the results and expand on some of the applications.

We thank the reviewer for the nice words about TRAILS, and we appreciate the comments and suggestions for improving the manuscript.

Particular points:

- p.6, Figure 2, Panel B: I do think that displaying the results here as bars that start at 0 is not helpful and unnecessary. It squeezes the whiskers that show the distribution around the mean, making it hard to compare some of them. My suggestion would be to just show a line for the mean of the estimates and whiskers, and the limits of the y-axis chosen to allow better comparison. Perhaps even points for each estimated value from a replicate. Related to this: This plot is based on 5 replicates. While general trends are definitely exposed by this, I do think a higher number of replicates would be better to reduce the random noise.

We have modified panel B in figure 2 to present the results in a clearer way:

- We have only included the n_AB = n_ABC = 1 and the n_AB = n_ABC = 5 cases, as they alone exemplify most of the results found by this analysis.
- Furthermore, we have changed the plot type, and we have plotted the values as (estimated-true)/true, to ease comparison.
- We have simulated 15 more replicates of each case to reduce random noise.

- p.8, Figure 4: This Figure demonstrates that TRAILS infers low coalescence times around a genetic variant under selection. I think it would be very helpful to supplement this with replicates of simulated 200kb neutral regions, and tally the distribution of the maximum posterior for those. While panel B) shows the theoretical expected value, and deviation from it, It is unclear how much the statistics vary around the mean under neutrality. Knowing this variance is necessary in empirical applications to assess significance of candidate regions. Thus, I think providing some sense of the variability would better exhibit the potential of the method for this application. Please mention here already that the interval boundaries are chosen such that the expected distribution is uniform.

We have updated fig. 4 by adding a total of 20 replicates for the selective sweep. We have also added the neutral case for comparison as an additional panel (fig. 4C). Finally, we have added in the caption that the “time intervals are adjusted so that all intervals have equal probability of observing a coalescent event”.

- p.9, l.191-201: In this paragraph, the estimates of the split times are provided as ranges. perhaps confidence intervals. It is unclear how these ranges are computed, since the preceding details describe how to obtain point estimates. Please provide details on whether these ranges are confidence intervals and how they are obtained. Through bootstrapping of the data? Using curvature of the likelihood surface?

Because the tip branch lengths are estimated separately for each species, there are several estimates for the branch lengths of the speciation tree. For the HC split, for example, 5.51 MYA corresponds to the human branch, while 5.81 MYA corresponds to the chimp branch.

In order to have a better idea about the variability of the estimates, we have performed parametric bootstrapping of the fitted model, and we have generated 95% confidence intervals of each parameter using fitted normals. Information about how this was performed can be found in the “Real

data" subsection of the "Methods summary" (and in section 10 of the Supporting Information). We now state in the text on page 9 that these are estimated 95% confidence intervals.

- p. 15, l. 357-262: While the details of the method are presented in the supplement, I think it would be good to provide a few more details about the computation of the transition probabilities in this section of the main text. Perhaps add a sentence like: "We can discretize the CTMC into a DMTC by evaluating it at the boundaries of the discretization intervals. This DMTC can then be used to compute discretized joint probabilities of the genealogies (hidden states) at the left and the right locus by considering the corresponding paths of the DMTC. The transition probabilities can then be obtained upon dividing by the discretized marginals."

We have added more details in the main text about the computation of the transition probability matrix under the "Transition probabilities of the HMM" section of the "Methods summary". We have also added a sentence reiterating that the full mathematical derivation is described in the Supporting Information.

- The runtime of the method is not mentioned for any of the analyses presented in the paper. To allow researchers interested in applying the method to judge the resources necessary to perform analyses, I think it is necessary to provide more details here. Please provide details on the runtime (and parallel architecture used) of analysing the simulated replicates for Figure 2B), estimating the parameters from the 50Mb HCGO-alignment presented in Figure 5A), and the posterior decoding presented in Figure 5B).

In order to get an idea about the most computationally expensive steps of the optimization procedure, we have benchmarked the computation of the transition probability matrix and the calculation of the likelihood. We have added this information in a new section of the Supporting Information (section 9), together with details about the runtime specifics for the optimizations performed in the manuscript.

- In the supplement, the state spaces and transition rates for the CTMC with 1,2, and 3 lineages are presented. However, were these obtained by manually enumerating all of them, or is there some structure underlying these that was used by the authors to enumerate them? The reason for this question is that if these are enumerated by hand, extending the method to 4 or more lineages will be very unwieldy, whereas if some structure of the problem can be used, extensions might be less cumbersome. If the authors have some insight into the structure of the problem, and perhaps some more general formulas, please present these.

One can obtain the full state space of the CTMC for an arbitrary number of sequences by recognising that the number of states corresponds to the even entries of the Bell number series. Once the full state space is enumerated and encoded properly (such as using minimally superincreasing integers to represent all uncoalesced and coalesced sites with a unique numeric identifier, as proposed in figure S6 and through the supplementary material), one can use an iterative approach to build the transition rate matrix by checking whether transitions through coalescence or recombination are allowed between pairs of states. We have included this explanation in the Supporting Information (section "Beyond three species") to make it clear how to extend the model for a larger number of

sequences. We have also included some R code (https://github.com/rivasiker/trails_paper/blob/main/state_space_exploration/state_space.qmd) for getting the state space and transition rate matrix for an arbitrary number of lineages (even though the current implementation is only feasible for n<6).

- The authors do present elegant approaches in the supplement to compute correct probabilities for the CTMC in cases where multiple coalescent events among the ingroup happen in the last "infinite" interval. However, it appears that t_upper is used as an upper bound for coalescent events among the ingroup when computing the emission probabilities. Is this correct? Are these transition and emission probabilities then combined in the HMM? While I do not think that this will majorly effect results if t_upper is large enough, I think this inconsistency should be highlighted (if it does exist).

The reviewer correctly points out that t_upper would limit the upper bound of the coalescence times for the deepest interval. However, t_upper is only used for the emission probabilities to be able to include an outgroup in the multiple genome alignment. In contrast, the transition probabilities are only calculated using three species without an outgroup, in order to avoid modelling complex ILS scenarios involving four sequences. Thus, an assumption of the model is that the divergence with the outgroup is large enough so that the majority of coalescent events have already happened before reaching the speciation event with the outgroup. We have added these details in section 6 of the supplementary material. Additionally, there is a reference to this assumption in the “Emission probabilities of the HMM” section of the “Methods summary”.

Minor points:

- p.5, l.112: a bound-constrained search algorithm that optimizes the likelihood function by evaluating it directly. [I think it would be good to state that no gradients or EM are computed.]

Done.

- p.5, l.132: Please clarify this statement. Why do these coalescent events cause underestimation?

It is difficult to pinpoint exactly why recombination is underestimated. We believe that some of the bias might be produced by recombination events that cannot be recovered from the sequence data, are thus missed and have the effect of underestimating the recombination rate. One of such scenarios involve recombination events that only cause small changes in the coalescence tree (for example, not changing the topology, and only changing the coalescent times by a few generations) and are thus almost impossible to detect from sequence data. We have modified this sentence to make it clearer.

- p.7, Figure 3, panel A: Please emphasize (perhaps in the caption) that 'first' and 'second' coalescence event refer to the order of events, thus it is possible that different extant lineages are coalescing at this 'first' or 'second' event at different loci.

Done.

- p.8, l.169: The signal observed in the posterior decoding can be summarized by comparing the proportion of sites with the maximum posterior probability in certain time intervals to the theoretical expectation.

Done.

- p.9, l.186: ..., choosing the parameter values estimated in Rivas-González as starting values ... [it is unclear what "this branch" refers to.]

Yes, this was misleading. We have removed the reference to “this branch”.

- p.9, l.188: The supplement states that other algorithms are possible, so state this here too.
Done.
- p.9, l.192: Please provide a reference for the value g=25 years.
According to Wang et al. 2023, the current human generation time is 26.9 years, while the chimpanzee generation time was estimated to be 24.63, and 19.3 in gorillas (Langergraber et al. 2012). We consider 25 to be a good compromise between these generation times. We have added these two references in the main text.
- p.10, l.225: Wouldn't it be more appropriate to have a time in years represent each interval and then take the weighted mean of that?
The reason for doing this is that the start and end points of the intervals are divided following the quantiles of an exponential distribution to ensure that a similar number of coalescent events occur within all intervals. In order to preserve this, we decided to assign integers instead. Alternatively, the same graph would be achieved if instead of the mean time in years, the mean time in log-years was used as a proxy.
- p.16, l.404: It is stated here that the Nelder-Mead algorithm is used for optimization. Previously, it was stated that the L-BFGS-B algorithm is used. Please clarify.
Both Nelder-Mead and L-BFGS-B were run, and Nelder-Mead showed better convergence than L-BFGS-B for already-optimized TRAILS runs.
- p.16, l.409: ... posterior decoding with the true parameters fixed. Done.

Supplement:
- p.2, l.55: ... and sit in different lineages. We added the clause “each with their own genealogical history” to this sentence.
- p.4, Figure S3: Add to caption: "Grey indicates the diagonal entries, which are computed as the negative of the sum of the off-diagonal entries in the corresponding row." Done.
- p.4, l.94: Please provide more details how the probabilities are mixed. Done.
- p.6, l.112: ... point t using \pi_{ABC}' = \pi_{ABC} exp(tQ_{ABC}). Done.
- p.5, l.115: Remove one period. Done.
- p.8, l.125: ... two topologies is known as incomplete lineage sorting … Done.
- p.9, l.141: Why is the number given by the Bell number series? Please provide an explanation or a citation. It is given by the Bell numbers because each state can be thought of as a possible configuration of a set with 2n elements, corresponding to all sites and lineages involved in the CTMC. A group of such elements correspond to either the left and the right site being in the same chromosome, or to two sites at either the left or the right side having coalesced. The state space of the CTMC is then defined as all possible partitions of the elements. We have added this information in section 2.4 of the Supporting Information.
- p.11, l.181: ... two-sequence CTMC, and, later, that lineage coalesces with … Done.
- p.12, l.187: Additionally, if the first coalescent event does not happen between … Done.
- p.16, l.286: Does this need to be F(t) = e^{tQ}? Yes, thank you for pointing this out.
- p.16, l.293 (and following equations): I believe the order of the matrix exponentials has to be reversed? Each next step has to be multiplied from the right. Thus, e^{rQ} should be the leftmost exponential, followed by (s-r), followed by (t-s). Similar with most other equations in the following sections. I might also be wrong about this. In this equation, t is the total time of the interval, s is the time from the first coalescent until the end of the interval, and r is the time from the second

coalescent until the end of the interval (see diagram below). Thus, the first interval, backwards in time, should be (t-s), followed by (s-r), and finishing with r.

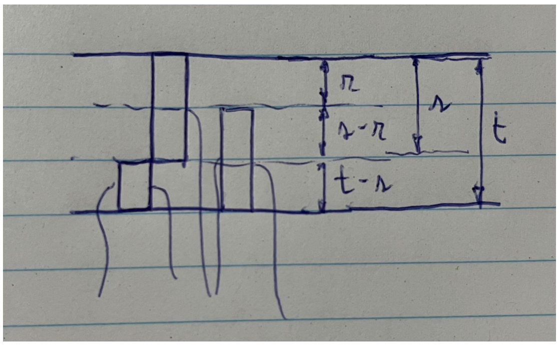

- p.17, l.325: ... we need to calculate infinite integrals of … Done.
- p.19, l.343: The states of the DTMC describe the marginal genealogical histories of the sequences. However, these states cannot be observed directly. Done.
- p.21, l.378: Should the rate of the exponential be the inverse of the ancestral population size instead of 1? Yes, but the computations are made in units of N_ABC, and everything is scaled accordingly.
- p.23, l.417: Pr(a_0) is not defined. Is it the stationary distribution of the mutation matrix? Pr(a_0) is the starting probability for the a_0 nucleotide. This corresponds to ¼ in the Jukes-Cantor model, as all nucleotides have the same initial probability. We have added this information in section 5.5 of the Supporting Information.

Reviewer #2: This is a nice paper and I enjoyed reading and thinking about it. The authors extend a previous approach to ancestral demographic inference from a multi-species genome sequence alignment, by introducing a more sophisticated representation of the coalescent process.

I don't have too many comments or suggestions to make, as it's a fairly self-contained methodological study and the manuscript motivates and describes the methods and approach well. I'm persuaded that this is a useful approach, and a potentially powerful framework for tackling problems in this area. The results on the great ape alignment provide a helpful demonstration of how it might be used.

There were just a few things I think the authors might address in a bit more detail, two of which relate to assumptions of the model.

We thank the reviewer for the kind words about the framework. The helpful comments and suggestions have helped us improve the paper.

Firstly, the model explicitly assumes that the outgroup is sufficiently remote that there is zero ILS between it and the A, B and C lineages involved in the focal speciation events. But at the same time it assumes that e.g. mutation and recombination rates have remained unchanged on all these lineages. In reality neither assumption might hold. But the ILS assumption seems particularly relevant. For example in the case of the HCGO divergence there will be about 13% ILS between HC, G and O using the parameters estimated in the paper. How does this impact the performance of the method or inferences drawn from it? Can it be mitigated in filtering the input alignment blocks? (I didn't see a discussion of this in the Methods.)

The reviewer correctly points out that based on the estimated parameters the proportion of ILS between HC, G and O would be around 13%, which violates one of the assumptions of TRAILS. As suggested, one solution could be filtering of alignment blocks where a HG or CG mutation is observed. However, these mutations are also produced by recurrent mutation of the same nucleotide in different branches of the phylogeny (e.g., an H mutation and a G mutation that happen independently).

The best way of overcoming this problem would be to model more complex ILS scenarios involving more than three species and an outgroup. This manuscript lays out the theoretical framework for achieving this. We have included this information in the last paragraph of the Discussion.

Secondly, TRAILS fits a 'clean split' speciation model in which there is no admixture between branches after their divergence. The authors discuss how the method might respond when there is potential departure from this in the data, in the context of the long V3 fragment in Fig. 5. One question which arises is whether there are more systematic approaches to detect such signals. For example, can one identify or quantify unexpectedly long fragments based e.g. on their posterior odds under the HMM? For having identified them, one could then look at the numbers of V3 and V2 topologies. Genome-wide asymmetry in these classes could be diagnostic of admixture or introgression, whereas the effects of selection if widespread might be expected to be symmetric. Did (or might) the authors investigate this?

Much information can be drawn from the posterior probability, including instances of genomic fragments that deviate from neutral expectations. Unidirectional introgression would be the easiest to characterize from the posterior decoding data, since, as the reviewer points out, it would create a genome-wide signal for an excess of one of the incongruent topologies with coalescent times that are closer to present and with fragment lengths that are longer than expected. One could perform a genome-wide scan, similar to what is proposed in figure 4B, to detect deviation from the expectation. In fact, a similar approach was applied in Rivas-González et al. (2023) (DOI: 10.1126/science.abn4409) to get a general genome-wide summary of the asymmetry between the ILS topologies. We agree that a more thorough investigation of the posterior decoding might reveal interesting patterns, but it merits a separate study analyzing empirical data in greater detail.

Thirdly, I think it is fair to say that the method is considerably more complex in terms of its underlying machinery than previous approaches such as CoalHMM. It would be good if the authors could comment on how this influences performance and scaling considerations, e.g. what are typical run times, memory requirements etc for the cases presented, particularly as one adds time intervals?

We have added the run times and practical considerations for running TRAILS in a new section of the Supporting Information (section 9). Here, we describe the general runtimes of the analyses performed in the manuscript, and we benchmark the computationally heavy steps of the optimization procedure (namely, the computation of the transition probability matrix and the calculation of the likelihood).

I noticed a couple of typos, in the equation on p. 9 and the preceding text, N_{ABC} should surely be N_{AB}.

Thank you for pointing this out. We have fixed the equation.

I liked the Supplement a lot; it provides a clear discussion which addressed most of the questions I had about the method and its implementation. I do think it will still be difficult to follow for anyone new to the ideas involved, but that's perhaps unavoidable. I have a couple of minor suggestions.

In discussing the basic CTMC, I wonder is it worth noting that there are two aspects of coalescence involved - one being the merging of homologous sequences at a particular locus (e.g. in going from \omega_{00} to \omega_{30} or \omega_{03}), and the other being the linking of two separate loci (e.g. in going from state 1 to another state in \omega_{00}). It's a minor thing but I think many readers will be more familiar with the first than the second. You might also consider reproducing the state diagram for the CTMC in the single-sequence case, to illustrate the process.

We have made clear that these are two different “coalescent” processes in the Supporting Information section 2.2.

The other suggestion is to change the red/blue colours in Fig S5, as there is potential confusion with other figures in which the same colours distinguish sequences/species.

We have changed the colors of fig. S3, S5 and S8 to avoid this potential confusion.

Aylwyn Scally

Reviewer #3: The ms by Rivas-Gonzalez et al. reports a novel powerful extension of the Coal-HMM framework published by part of the authors some years ago. The ms also advertises for TRAILS, the newest release of a series of softwares that infer ancestral population sizes, speciation times and recombination rates for a genomic alignment of 3 species, plus an outgroup. The study assesses the power and the limitations of the method (and the related software) with great care, using simulations and a human-chimp-gorilla+orang-utan alignment.

I would like first to thank the authors for the care they took to explain the method in a very clear and comprehensive supplementary material. With only basic knowledge of HMM and coalescent theory, it is quite easy to follow, enlightening and enjoyable. It helped me a lot having a better grasp of what was at stake and improved a lot my comprehension of Coal-HMM techniques. Thank you.

More generally, the ms is scientifically sound, easy to read and quite convincing. I have only a list of comments and suggestions that may help to produce an even better/clearer article.

We appreciate the nice words about the manuscript and the constructive comments and suggestions.

1) My first and most important suggestion is to change your strategy for the figures 2-5 of the main text. As they are currently, they are quite difficult to read and even more to understand. I suspect that you were tempted to provide as much info as you could, but in the end, a casual reader such as my poor self can suffer from the impression of being overwhelmed by the generosity of the figures. I shall now detail few more concrete suggestions, figure by figure.

Firstly, we want to thank the reviewer for their many suggestions regarding the figures that have helped improve the readability and interpretability of results.

- Figure 2. Panel A, what is t_upper? Wouldn't a t_3 ranging from AB to ABC be more intuitive? Or maybe there is something I don't get (probably).

t_upper is the time between the start of time of the last interval and the speciation time between ABC and the outgroup. t_upper is only used for the emission probabilities, since the transition probabilities are calculated without the need of an outgroup species. Also, it is assumed to be large. We have added details about this to Supplementary Information Section 6.

We agree that t_3 would be more intuitive, but the reason why we have parameterized the model to estimate t_upper instead of t_3 is that the cutpoints of the intervals deep in time (in the ABC ancestor) are parameterized by N_ABC, and, thus, when the HMM is being optimized, the value assumed for t_3 might be smaller than the start time of the last interval. This is impossible, since the model assumes that all the interval cutpoints for ABC are contained between the speciation time between AB and C, and the speciation time between ABC and the outgroup. Thus, the choice of t_upper instead of t_3 is purely for practical optimization purposes.

On panel B, I am not sure whether having both n_{AB}=3 and n_{AB}=3 is really helping. Reducing the number of subplot can only make the figure clearer. In the current format, it is too crowded and fonts are too small for the readers. Furthermore, why don't you use whisker-plots instead of bar-plots?

We have modified panel B in figure 2 to present the results in a clearer way. Please, refer to reviewer #1's first major point for further details.

- Figure 3. I wonder whether the names "V0" to "V3" is a better choice than newick self-explanatory strings such as "((A,B),C)"? Furthermore the posterior probabilities within the heatmaps are not well contrasted so it is not visually convincing that the HMM does a good job (with the exception of the topology). Did you try using log(prob) for the color code? or less categories ? Furthermore, the tree with tiny slices denoted by Ss and Fs is way to small to be read and again not very helpful. Please make it more straightfoward.

- The reason why the names “V0” to “V3” were chosen was that V0 and V1 have the same topology, corresponding to ((A,B),C), but the first coalescent time backwards in time falls within the speciation events for V0, but happens deeper than the second speciation event for V1. Moreover, the V0-V3 labels correspond directly with those used in the Supporting Information, where concise labeling is needed to ease mathematical formulations. Thus, we have decided to keep the current labeling scheme. For clarity, we edited the text where V0 and V1 are first discussed.
- Based on the reviewer's suggestions, we have tried several other color schemes and data transformations. However, we have decided to retain the original color scheme because
    - Figures 3, 4A and 5C have the same color scale, which eases comparison across figures.
    - TRAILS does a better job at recovering the topology than the coalescent times, which is reflected in the posterior probability. One could use a different color scale for each of the panels in fig. 3 (e.g., decreasing the limits of the color scale or log-transforming the probabilities), but keeping the same scale across panels showcases exactly what uncertainty to expect for each of the features.
- Using fewer categories does not improve the readability of the figure, since there is not enough resolution to see smaller changes in the genealogies.
- We believe that the tree with the slices is necessary to showcase the different intervals used in the posterior decoding, since they are different for the first and the second coalescent. We have, however, increased the font sizes to improve readability.

- Figure 4. Same remark for the colors of heatmaps. It is not obvious how the Posterior max is computed. Finally, theoretical expectations means theoretical NEUTRAL expectations, correct?

We have updated fig. 4 to make our point clearer. Instead of using the posterior max (which was defined as, for each time interval, the proportion of sites for which the maximum posterior probability falls within that time interval), we are using the mean posterior probability per interval. We have also increased the number of replicates for the selected and neutral cases, to have an idea about the variability.

Yes, the theoretical expectation is in the neutral case. We have added this information to the text and captions.

- Figure 5. Panel B has remained opaque to me (I have abandoned, even as a reviewer). Here again, recalling what "V0" and others are implies going back and forth between Figure 5 and Figure 2. I again believe the newick strings is a better choice than V[0-3]. Like in general, font sizes and plots are too small.

We have increased the font size for fig. 5, and change the ordering of the panels to improve readability. We have also included the correspondence of the V0-V3 labeling and the topology of the tree in Newick format in the caption of the figure.

2) The underestimation of rho (figure 2 and l131) is intriguing. As the convergence of the other estimates is good, or even very good, this is mind-bugging. The authors suggest that it stems from rapid coalescence after recombination that results in undetectable recombination events. But really what intuitively matters is the occurrence of mutations in the time lag between both types of events. In this case, tuning the \mu to \rho ratio would change the strength of the bias, lowering it as it increase. More generally, as it is the only poor estimate, I recommend explore different strategies to overcome the bias or at least better characterizing the issue and discussing it more.

The estimation of the recombination rate is definitely the most challenging, and the source of the bias seems to be different from that in the rest of the parameters, as a more complex model does not make its estimation significantly better. As part of answering reviewer #1's second minor point, we have rewritten this paragraph to make our point clearer. As correctly pointed out by the reviewer, we believe that the source of the bias is that TRAILS is incapable of observing enough mutations for detecting short fragments of ancestry, especially when the recombination event changes the tree in small ways (e.g., topology is not changed and coalescent times are changed by a few generations). This has the effect of underestimating the recombination rate, as some events are missed.

3) The authors provide an estimate of the parameter from a single region of chromosome 1. Having few regions from the same and from different chromosomes and comparing the results is certainly a good move. I somehow have a vague memory that one of the chromosomes had a different pattern of ILS, but I may be totally wrong (this an old memory).

We agree that a more comprehensive investigation of the data could reveal interesting patterns and differences among chromosomes. For example, the X chromosome in the human-chimp-gorilla case shows large regions devoid of ILS (see Dutheil et al. 2015, 10.1371/journal.pgen.1005451), and TRAILS can now pinpoint exactly where coalescence of these regions happened in the speciation tree. However, we believe that a detailed exploration of empirical data merits a separate study.

4) Reports of CPU time and memory consumption are lacking. Especially discussing them regarding the differences with previous simpler versions. In general, it is interesting to know how much CPU resource (e.g. carbon) we spend for how much precision we gained. What did we gain for what cost? About CPU time, the complexity is likely linear with alignment size, but how is it with number of time categories in AB and in ABC.

We have added information about the run time in the manuscript, more specifically under a new subsection 9 in the Supporting Information.

:: A collection of minors remarks ::

l163 : this approximation is not very good. A better one is "2ln(2Ns + c)/s" where c is the Euler constant. At least better use 2Ns than 2N.

We have updated the formula with equation A17 in Herminsson and Pennings 2005.

l199: insert "new" --> "our NEW estimates" We have added “parameter” instead of “new”.

l269-l278: can we see the xy-plots of the correlated variables (probably in the supp)?

Using parametric bootstrapping, we have calculated correlation coefficients for every pair of parameters. We have added a new section 10 in the Supporting Information describing the procedure and the results.

- figure 5C and l257-291. Any temptation to compute some kind of standard neutrality test (e.g. dn/ds on the coding, branch length inflation, or SFS-based using publicly available polymorphisms for this locus)?

LDLRAD1 does not show signals of positive selection in hominids (based on dN/dS from Rivas-González et al. 2023). Additionally, this gene is not particularly strongly constrained in primates, as measured by PhastCons (Siepel et al. 2005) or PhyloP (Pollard et. al 2010). We have added this information in the text.

- l 321 & l56 (supp): please state that the coalescent occurs between the two ancestors that were carrying the left and right ancestral materials. I did get it, but at first I was disturbed.

We have made this clearer in the main text (“Methods summary”) and the supplement Supporting Information (section 2.2).

- l368 : "ILS can be neglected" instead of "no ILS". Done.

- l429: the second "forward" should be replaced by "backward" Done.

- l112 (supp) \pi_{AB} should be \pi_{ABC} Done.

- l115 (supp) ".." -> "." Done.

- p9-10 (supp). I guess the matrices were recoded in sparse format, which really speed up calculation and reduce memory. Yes, this is automatically achieved using numpy, which always uses the most efficient representation of matrices.

- l286 (supp). I am not sure but I think it should be exp(tQ) and not exp(tA) Yes, thank you for pointing it out.

- l473. This "e" is different from the (2,1) vector described earlier l265, no? They are both vectors where all of the entries are ones (although they have different sizes). We have added this information to clarify it.

- l481. It is not obvious to me how it works as B is a matrix and e a vector (from what I can understand). B is a matrix, but B(o_i) is column in the emission probability matrix corresponding to the state emitted at the i'th observation. Thus, B(o_i) is also a vector.
